# The impact of an additional implant under the saddle of removable partial dentures in Kennedy Class II edentulism on oral health-related quality of life and oral function: a case series report

**DOI:** 10.1186/s40729-022-00463-x

**Published:** 2022-12-01

**Authors:** Toshifumi Nogawa, Yoshiyuki Takayama, Makoto Ishikawa, Atsuro Yokoyama

**Affiliations:** 1 Department of Preventive Dentistry, Hokkaido University Hospital, Kita 14, Nishi 5, Kita-Ku, Sapporo, 060-8648 Japan; 2 Department of Removable Prosthodontics, Hokkaido University Hospital, Sapporo, Japan; 3 Clinic of Oral Implants, Center for Advanced Oral Medicine, Hokkaido University Hospital, Sapporo, Japan; 4 Department of Oral Functional Prosthodontics, Division of Oral Functional Science, Faculty of Dental Medicine, Hokkaido University, Sapporo, Japan

**Keywords:** Implant-supported partial dentures, Removable partial dentures, Masticatory performance, Oral health-related quality of life, Satisfaction

## Abstract

**Background:**

Implant-supported removable partial dentures (ISRPDs) provide effective prosthodontic treatment for partially edentulous patients. ISRPDs offer greater patient satisfaction and better oral function compared with removable partial dentures (RPDs) by enhancing denture stability and support. However, few clinical studies have focused on RPD design in patients with mandibular Kennedy Class II edentulism. The aim of this case reports was to investigate the oral function, oral health-related quality of life, and satisfaction of four patients with unilateral distal-extension mandibular RPDs with the same design which were replaced with ISRPDs. In addition, we investigated how each patient’s evaluation varied with the change from RPD to ISRPD.

**Case presentation:**

Four patients had unilateral distal-extension mandibular edentulism and were missing the first and second molars and the first and second premolars. They received one implant (4.0 mm in diameter, 8.0 mm in length; IAT EXA PLUS Bone level; Nippon Piston Ring Co. Ltd, Saitama, Japan) at the position equivalent to the first molar in the edentulous residual ridge perpendicular to the occlusal plane. Implant position was determined by surgical guide plate. RPDs were fabricated after the residual mucosal membrane had healed. The basic design of the RPD was as follows: a cobalt–chromium alloy cast metal framework denture with a lingual bar as the major connector, a double Akers clasp on the molars and an auxiliary retainer on the premolar as indirect retainers, and a wrought wire clasp and a cast cingulum rest (combination clasp) as direct retainers. Masticatory performance, occlusal force, oral health-related quality of life, and satisfaction were estimated at baseline, and at time points after insertion of the RPD and after insertion of the adapted ISRPD. Each evaluation item showed a tendency for improvement on insertion of the new RPD. Masticatory performance and satisfaction tended to be better after insertion of the ISRPD than after insertion of the RPD.

**Conclusion:**

Our findings suggest that ISRPDs provided better patient satisfaction and masticatory performance than RPDs in patients with mandibular Kennedy Class II edentulism.

*Trial registration* UMIN Clinical Trials Registry and Japan Registry of Clinical Trials, UMIN000025283 and jRCTs012180003. Registered 19 February 2016 and 17 December 2018, https://www.umin.ac.jp/ and https://jrct.niph.go.jp/

## Background

Many previous studies have reported on the effectiveness of implant-supported removable partial dentures (ISRPDs) for partially edentulous patients. According to a review and meta-analysis by Park et al. [1], ISRPDs significantly improved patient-reported outcome measures such as oral health-related quality of life (OHRQOL), satisfaction, and masticatory performance when compared with conventional removable partial dentures (RPDs) in patients with mandibular Kennedy Class I edentulism. Murakami et al. [2] reported that patients with free-end missing dentition in either the upper or lower jaw, and who were fitted with bilateral and unilateral implants with ISRPDs, exhibited increased occlusal force and improved masticatory efficiency in comparison with those with conventional RPDs. Distal-extension RPDs in function show complicated movement patterns with various combinations of pitching, rolling, yawing, and depression around the abutment teeth. Dental implants under unilateral distal-extension RPDs are considered to be effective in suppressing these movements. However, few studies have investigated this improved effectiveness in patients with mandibular Kennedy Class II edentulism [2, 3]. Additionally, most studies do not have a common design concept for the retainers of the ISRPDs.

Therefore, the aim of this pilot case report was to investigate masticatory performance, occlusal force, OHRQOL, and satisfaction in patients with unilateral distal-extension mandibular edentulism who have been fitted with ISRPDs with the same RPD design concept in a small sample. In addition, we investigated how each patient’s evaluation varied when changing from an RPD to an ISRPD.

## Case presentation

### Subjects

Four patients (all female) with a mean age of 70 ± 7.5 years who had unilateral distal-extension mandibular edentulism and were missing the first and second molars and the first and second premolars received dental implants and ISRPDs at the department of Removable Prosthodontics, Hokkaido University Hospital, from December 2016 to March 2021. None of the patients had complete maxillary dentures, temporomandibular disorders, xerostomia, poor oral hygiene, or any serious systemic diseases. All patients had previous mandibular denture before registration in this study.

The following information was collected for each patient: age, sex, number of remaining teeth and occlusal support, and Eichner classification. We obtained the patients’ information, masticatory performance, occlusal force, OHRQOL, and satisfaction at baseline (BL) (i.e., before insertion of the RPDs). The patients’ BL information is shown in Table 1.

**Table 1:** Characteristics of patients

Case	Sex	Age	Implant position	Remaining teeth	Occlusal support			Eichner classification	Opposing statement
					Full-arch	Anterior	Posterior		
1	Female	72	36	18	5	5	0	B4	Natural teeth and RPD
2	Female	60	46	17	5	4	1	B3	Natural teeth
3	Female	70	46	14	5	5	0	B4	Natural teeth and RPD
4	Female	78	36	14	4	2	2	B2	Natural teeth
Mean (SD)		70 (7.5)		15.8 (2.1)	4.8 (0.5)	4 (1.4)	0.8 (1.0)		

*RPD* removable partial denture

**Fig. 1:**
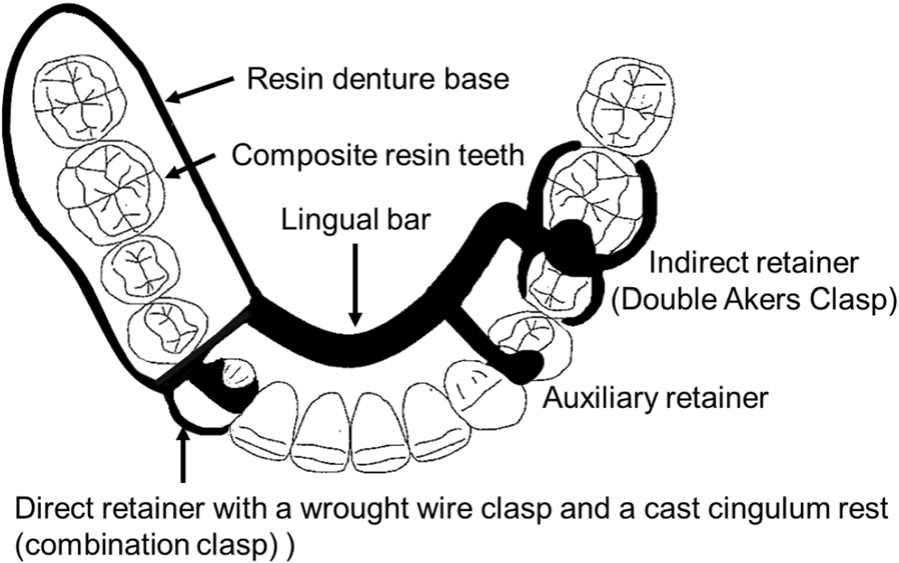
Basic design of removable partial denture

**Fig. 2:**
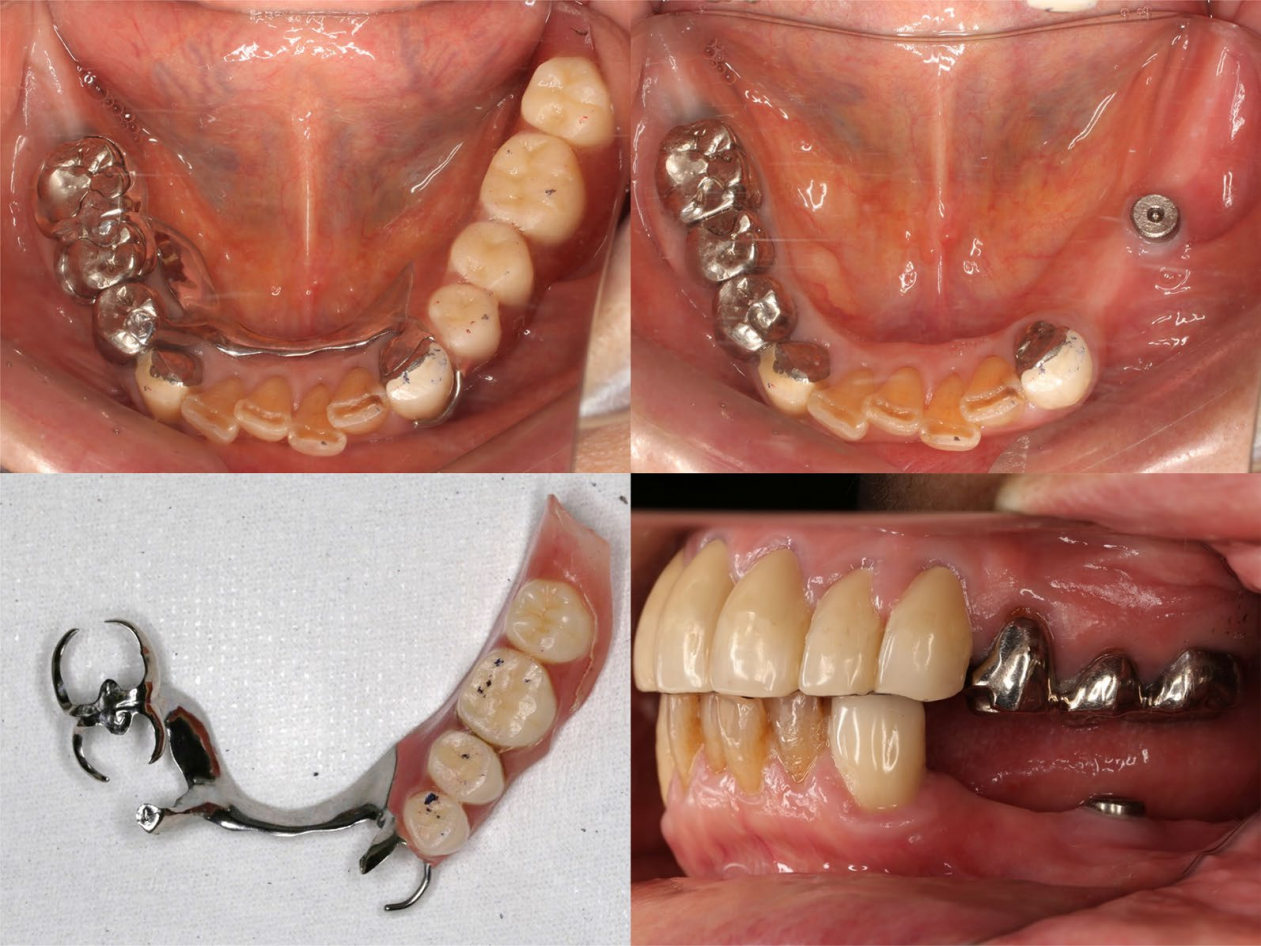
Intraoral views of Case 1 with implant-supported removable partial denture in place

Each patient received an implant (4.0 mm in diameter, 8.0 mm in length; IAT EXA PLUS Bone level; Nippon Piston Ring Co. Ltd, Saitama, Japan) at the position equivalent to the first molar in the edentulous residual ridge perpendicular to the occlusal plane. Implant position was determined by surgical guide plate. The RPDs were fabricated after the residual mucosal membrane had healed. The basic design of the RPD is shown in Fig. 1 [4]. The cobalt–chromium alloy cast metal framework denture consisted of a lingual bar as the major connector, with a double Akers clasp on the molars and an auxiliary retainer on the premolar as indirect retainers, and a wrought wire clasp and a cast cingulum rest (combination clasp) as direct retainers (Fig. 2).

At 3 months after insertion of the RPD (R1), masticatory performance, occlusal force, OHRQOL and satisfaction were measured. A second surgery was then carried out and a 5.0-mm-high healing abutment was connected to the implant body. The denture base of the ISRPD was relined to contact with the top of the healing abutment with acrylic resin (Unifast III; GC Corp., Tokyo, Japan), and relieve around abutment. The healing abutment served a role in supporting the denture. At 3 months (IS1), 6 months (IS2) and 12 months (IS3) after fitting the ISRPD, masticatory performance, occlusal force, OHRQOL and satisfaction were again measured.

Additionally, the information on adverse events from implant placement to the end of the observation period, 30th September 2022, was collected.

All time points were compared using the Wilcoxon signed rank test about every evaluation. All statistical analyses were performed using JMP^®^ 16.2.0 (SAS Institute Inc., Cary, NC, USA) with a significance level of 0.05.

### Patients’ reported outcomes

The patients’ reported outcomes were recorded for OHRQOL and the degree of satisfaction with the treatment.

OHRQOL was measured using the Oral Health Impact Profile (OHIP) [5, 6]. This questionnaire measures the patient’s perception of the impact of oral disorders on their well-being using 49 questions. The patient’s response to each question was recorded using a 5-point scale with a maximum score of 196 points.

Satisfaction with esthetics, pain, comfort, stability, speaking, and mastication as well as general satisfaction was measured using a 100-mm linear visual analog scale (VAS) [7].

### Oral function

Oral function (masticatory performance) was assessed by using a gummy jelly and measuring occlusal force with a pressure-sensitive sheet.

The patients were asked to chew a gummy jelly (Glucolumn; GC Corp) for 20 s [8]. They were then asked to rinse their mouth with 10 mL of distilled water and spit into a cup. The gummy jelly and saliva within the cup were then filtered, and the filtrate was collected. The glucose concentration in the filtrate was measured as the glucose extraction using a glucose testing device (GlucoSensor GS-II; GC Corp). The filtrate was measured three times for the right, left, and both sides, respectively, and the average values were used for the analysis. The mean of the measurements was used as an index of masticatory performance.

Occlusal force was measured with a pressure-sensitive sheet (Dental Prescale 50H type R; GC Corp). The patients sat in a dental chair with their occlusal plane parallel to the floor. The sheet was placed along the maxillary dentition. They were instructed to clench on the sheet with maximum force in the intercuspal position for 3 s. Then, the occlusal force was measured with a computerized imaging scanner (Occluzer FPD-705; FUJIFILM Corp, Tokyo, Japan). The occlusal force test was performed three times, and the mean of the results was used for the analysis [9].

### Approval and consent

Written informed consent was obtained from the patients for publication of this study. The study protocol was approved by Hokkaido University Certified Review Board (018-001, jRCTs012180003).

## Variation of evaluations (Tables 2, 3, 4, 5, 6, 7, 8)

**Table 2:** Complications of ISRPDs

Case	Observation period (months)	Tooth region		Complication	
		Months			
1	57	LL3	Decementation at metal core		Denture fracture at implant
			10		20
2	36	n.p			
3	33	UL3	Extraction for root fracture	LR3	Extraction for root fracture
			19		28
4	34	n.p			

*LL* lower and left, *LR* lower and right, *UL* upper and left

The observation period for complications was from the insertion of ISRPD until September 2022

n.p.: no problem

**Table 3:** Variation in masticatory performance of each patient

Masticatory performance (mg/dL)															
Case	Both sides					Prosthesis side					Natural dentition side				
	BL	R1	IS1	IS2	IS3	BL	R1	IS1	IS2	IS3	BL	R1	IS1	IS2	IS3
1	149.0	168.5	171.5	173.5	178.5	176.5	165.5	193.0	201.5	197.0	145.0	142.0	169.0	189.5	164.0
2	73.5	120.5	132.0	118.5	138.0	70.0	107.0	125.5	136.5	161.0	52.0	145.0	154.5	138.5	167.0
3	184.5	135.0	201.5	163.0	188.0	90.5	150.5	229.0	158.0	178.0	194.5	122.5	130.5	136.5	131.5
4	142.5	163.5	143.0	163.0	164.0	64.0	122.5	130.5	137.5	128.5	112.0	139.5	127.0	170.0	144.5
Mean	137.4	146.9	162.0	154.5	167.1	100.3	136.4	169.5	158.4	166.1	125.9	137.3	145.3	158.6	151.8

*BL* baseline, *R1* 3 months after insertion of removable partial denture (RPD), *IS1* 3 months after insertion of implant-supported RPD, *IS2* 6 months after insertion of implant-supported RPD, *IS3* 12 months after insertion of implant-supported RPD

**Table 4:** Variation in occlusal force of each patient

Occlusal force (N)															
Case	Full-arch					Prosthesis side					Natural dentition side				
	BL	R1	IS1	IS2	IS3	BL	R1	IS1	IS2	IS3	BL	R1	IS1	IS2	IS3
1	603.0	585.7	516.0	431.0	447.1	219.6	334.3	288.8	207.0	204.3	383.4	251.4	227.2	224.0	242.9
2	190.2	373.6	276.6	333.9	335.7	42.8	81.8	109.1	131.2	141.7	147.4	291.8	167.5	202.7	194.0
3	247.0	368.6	345.0	252.6	168.7	123.5	171.1	104.7	100.1	60.1	123.5	197.5	202.2	152.7	108.6
4	293.4	326.5	356.3	527.9	377.0	113.4	126.6	183.2	284.6	209.6	180.0	199.9	161.0	243.4	167.5
Mean	333.4	413.6	373.4	386.4	332.1	124.8	178.5	171.5	180.7	153.9	208.6	235.1	189.4	205.7	178.2

*BL* baseline, *R1* 3 months after insertion of removable partial denture (RPD), *IS1* 3 months after insertion of implant-supported RPD, *IS2* 6 months after insertion of implant-supported RPD, *IS3* 12 months after insertion of implant-supported RPD

**Table 5:** Variation in distribution of occlusal force on the prosthesis side

Distribution of occlusal force on the prosthesis side (%)					
Case	BL	R1	IS1	IS2	IS3
1	36.4	57.1	56.0	48.0	45.7
2	22.5	21.9	39.5	39.3	42.2
3	50.0	46.4	30.4	39.6	35.6
4	38.7	38.8	51.4	53.9	55.6
Mean	37.4	43.1	45.9	46.8	46.3

*BL* baseline, *R1* 3 months after insertion of removable partial denture (RPD), *IS1* 3 months after insertion of implant-supported RPD, *IS2* 6 months after insertion of implant-supported RPD, *IS3* 12 months after insertion of implant-supported RPD

**Table 6:** Variation in OHIP-49 of each patient

OHIP-49																									
Case	Four-factor																				Summary scores				
	Psychosocial impact					Oral function					Oro-facial appearance					Oro-facial pain									
	BL	R1	IS1	IS2	IS3	BL	R1	IS1	IS2	IS3	BL	R1	IS1	IS2	IS3	BL	R1	IS1	IS2	IS3	BL	R1	IS1	IS2	IS3
1	2	2	4	3	9	17	7	9	13	13	0	0	4	1	0	8	11	14	13	13	38	27	41	40	42
2	5	11	7	1	8	12	13	12	9	9	6	3	4	5	3	2	3	4	1	3	32	38	33	20	30
3	20	19	18	28	18	13	13	9	14	16	6	7	7	7	7	10	8	1	8	9	59	58	41	76	59
4	37	1	0	0	0	24	6	6	5	5	23	6	2	1	0	7	3	0	1	0	111	23	15	10	11
Mean	16	8.25	7.25	8	8.75	16.5	9.75	9	10.25	10.75	8.75	4	4.25	3.5	2.5	6.75	6.25	4.75	5.75	6.25	60	36.5	32.5	36.5	35.5

*BL* baseline, *R1* 3 months after insertion of removable partial denture (RPD), *IS1* 3 months after insertion of implant-supported RPD, *IS2* 6 months after insertion of implant-supported RPD, *IS3* 12 months after insertion of implant-supported RPD

**Table 7:** Variation in patient satisfaction

Satisfaction (VAS score)																				
Case	Esthetics					Mastication					Speaking					Pain				
	BL	R1	IS1	IS2	IS3	BL	R1	IS1	IS2	IS3	BL	R1	IS1	IS2	IS3	BL	R1	IS1	IS2	IS3
1	100.0	100.0	100.0	97.8	98.9	100.0	100.0	96.7	98.9	98.9	25.0	94.0	80.0	75.6	57.4	100.0	98.0	83.3	77.8	79.8
2	56.7	69.5	71.1	78.9	69.2	66.7	68.4	63.3	75.6	72.5	37.8	60.0	63.3	76.7	71.4	88.9	93.7	75.6	93.3	95.6
3	84.4	90.0	82.4	85.7	72.7	46.7	54.4	91.2	84.6	56.8	85.6	86.7	91.2	86.8	70.5	27.8	54.4	94.5	89.0	43.2
4	2.1	89.0	95.6	92.3	86.4	11.7	79.1	81.1	86.8	81.8	8.5	92.3	95.6	95.6	92.0	69.1	95.6	97.8	95.6	83.0
Mean	60.8	87.1	87.3	88.7	81.8	56.3	75.5	83.1	86.5	77.5	39.2	83.2	82.5	83.7	72.8	71.5	85.4	87.8	88.9	75.4

*BL* baseline, *R1* 3 months after insertion of removable partial denture (RPD), *IS1* 3 months after insertion of implant-supported RPD, *IS2* 6 months after insertion of implant-supported RPD, *IS3* 12 months after insertion of implant-supported RPD, *VAS* visual analog scale

**Table 8:** Variation in patient satisfaction of each patient

Satisfaction (VAS score)															
Case	Comfort					Stability					General satisfaction				
	BL	R1	IS1	IS2	IS3	BL	R1	IS1	IS2	IS3	BL	R1	IS1	IS2	IS3
1	95.0	100.0	100.0	93.3	78.7	95.0	100.0	97.8	87.8	98.9	100.0	98.0	97.8	96.7	95.7
2	71.1	54.7	64.4	64.4	68.1	72.2	70.5	64.4	70.0	68.1	73.3	65.3	68.9	76.7	68.1
3	30.0	55.6	95.6	86.8	40.9	27.8	56.7	89.0	85.7	40.9	28.9	57.8	96.7	94.5	52.3
4	17.0	91.2	98.9	89.0	95.5	25.5	89.0	97.8	87.9	93.2	4.3	94.5	98.9	95.6	94.3
Mean	53.3	75.4	89.7	83.4	70.8	55.1	79.1	87.3	82.9	75.3	51.6	78.9	90.6	90.9	77.6

*BL* baseline, *R1* 3 months after insertion of RPD, *IS1* 3 months after insertion of implant-supported RPD, *IS2* 6 months after insertion of implant-supported RPD, *IS3* 12 months after insertion of implant-supported RPD, *VAS* visual analog scale

The results for each evaluation item indicated an improvement with the insertion of the new RPD. Masticatory performance was better after the ISRPD was fitted than after the initial insertion of the RPD. The improvement in masticatory performance was greater on the prosthesis side than on the natural dentition side. Masticatory performance remained stable between R1 and IS3.

Occlusal force varied greatly due to individual differences, and the effect of fitting the ISRPD could not be clearly shown. However, when Case 3 was excluded, the ISRPD improved occlusal balance on both sides.

In Case 4, the OHIP-49 summary score improved from 111 to 23 points on insertion of the RPD. In the other cases, there was little change in the scores; the mean values revealed a tendency for improvement, but the influence was less than that in Case 4.

All satisfaction evaluation scores improved with the insertion of the RPD. Scores for esthetics, speaking, and pain remained much the same with the change from RPD to ISRPD, while the other scores improved. Satisfaction levels at IS3 were maintained from R1 to IS3, except in Case 3.

There was no significant difference among the time points for masticatory performance, occlusal force, OHIP scores, and VAS scores due to the extensive individual differences. In addition, masticatory performance, occlusal force, OHRQOL and satisfaction tended to improve with the insertion of the new RPD, but no significant change was observed after fitting of the ISRPD. However, the masticatory performance on the prosthetic side tended to improve with the insertion of the ISRPD.

Swelling after surgery of located implant occurred in all cases, but all healed. In addition, there was a case in which medication was required due to swelling of the surrounding gingiva after insertion of the healing abutment in Case 1. Loosening of healing abutments were occurred in Case 1 and 4.

All patients were cured by adjusting the dentures, while they complained of discomfort and pain at insertion of new dentures. Adverse events after ISRPD insertion are shown in Table 2.

## Discussion

This study was a pilot case study in patients with mandibular unilateral distal-extension edentulism (Kennedy Class II) to investigate the effect of the treatment with RPDs and ISRPDs on patients’ reported outcomes and oral function in a small sample.

Because many previous ISRPD studies [10, 11] have studied patients with bilateral distal-extension partial edentulism (Kennedy Class I), this study evaluated patients with mandibular Kennedy Class II edentulism with removable partial dentures with the same concept design. We previously reported that an ISRPD with a combination clasp distributes the load along the direct abutment tooth axis in the apical direction and suppresses displacement in the buccal direction compared with other types of retainers. In this clinical study, the ISRPD was designed accordingly [4].

There was no statistically significant difference in the treatment effect between the RPD and the ISRPD. However, OHRQOL at the 3 months after the insertion of the RPD or ISRPD tended to be higher than at the baseline. This improvement in OHRQOL could be accomplished by wearing a properly designed RPD with sufficient support, retention, and stability using a metal framework. It is reported that the minimal clinically important difference is 14 points in the OHIP-49 [12]. The variation in the OHIP-49 scores in this study was less than 14 points. Under the conditions of this study, OHRQOL might be less affected by the dental implant placed under the denture base to enhance support.

However, one study [1] reported that OHRQOL was improved by placing a dental implant in the edentulous space in patients with Kennedy class I edentulism. Further data are required to determine whether the difference in the results of this study is due to the difference in the type of partial edentulism or the denture design.

In terms of patient satisfaction, all outcomes after treatment with an RPD tended to be better than those before treatment. RPDs and ISRPDs tended to achieve similar results for speaking, esthetics, and pain. For these outcomes, the effect of strengthening the denture support with dental implants might be small. Additionally, patient satisfaction in terms of mastication, comfort, and stability tended to be better in ISRPDs than in RPDs. These factors would be directly influenced by suppressed denture movement by a dental implant in the edentulous space.

Masticatory performance tended to improve after RPD insertion in comparison with the that at baseline, and improved even further after ISRPD insertion. The improvement of masticatory performance was greater on the edentulous side. It is highly probable that denture movement was suppressed by the dental implant in the edentulous space, which contributed to the improved function [2, 3].

Occlusal force tended to be a higher after insertion of a new RPD than at baseline. The occlusal force on the natural dentition side also tended to decrease from R1 to IS3, but there was no significant difference among the time points. Additionally, taking the mean values into account, the amount of change in the occlusal force was not large, and the balance of occlusal force between the natural dentition side and the prosthesis side would be practically equal. Murakami et al. [2] reported that the occlusal force was greater on dentures with a supporting dental implant than on those without an implant. Additionally, they reported that there was no significant change in the occlusal force in the natural dentition area when a supporting dental implant was present. Therefore, we conclude that a dental implant under the denture base enhances occlusal support, suppresses movement of the denture base, and improves the occlusal balance between the two sides. However, because occlusal force is dependent on individual differences, future studies should be conducted with more patients.

In this case report, dental implant was inserted at the part corresponding to the first molar position. It is not clear how the implant position affects clinical effects in Kennedy Class II. There is the experimental study that the distally implant position suppresses the movement of the denture, and the medially implant position suppresses the movement of the abutment tooth [13, 14]. In this case study, the implant position was determined primarily to suppress denture movement. Since the behavior of dentures changes depending on the position, it might affect clinical results, but we believe that further investigation will be necessary in the future.

The weighted mean cumulative survival rate of the implants with ISRPDs in mandibular Kennedy Class I was reported at 96.60% (95% CI = 92.97–98.79) [15]. There was no severe event such as implant loss in this case report. However, there was loss of abutment tooth in Case 3. There were a few reports about survival rate of the abutment teeth in ISRPDs. The survival rates of abutment teeth at the ISRPD ranged from 79.2% to 100% after observation periods of 1–12.2 years [16]. It is reported that the survival rates over 5 years and 10 years were 93.0% and 89.7% in RPD patients [17]. It was possible that there was no difference in the prognosis of abutment teeth between ISRPDs and RPDs. In this study, one of four patients had the abutment tooth extracted. It suggested that the protective effect of implant support on the abutment tooth might be limited. However, it was a small case reports in a short observation period. It is necessary to further investigate the prognosis of the abutment teeth in ISRPDs.

### Limitations of this study

This study was a pilot case report with a small sample size for a large-scale interventional clinical trial. Because clinical evidence for ISRPDs is still lacking, a pilot study was necessary before proceeding with future clinical trials and experimental studies.

There was no statistically significant difference in patient-reported outcomes and oral function between RPDs and ISRPDs in this study. Although it is difficult to generalize the results of this study, our findings indicate a tendency towards improvement in patient-reported outcomes and oral function with ISRPDs in patients with mandibular Kennedy Class II edentulism. These findings may be useful as a reference for clinical studies of ISRPDs in patients with unilateral partial edentulism.

## Conclusions

The results of this case report suggest that patients with mandibular Kennedy Class II edentulism experienced greater satisfaction and improvement in masticatory performance with ISRPDs than with RPDs.

## Data Availability

The datasets used or analyzed during the current study are available from the corresponding author on reasonable request.

## Abbreviations

RPD: Removable partial dentures
ISRPD: Implant-supported removable partial dentures
